# Deciphering the trophic interaction between *Akkermansia muciniphila* and the butyrogenic gut commensal *Anaerostipes caccae* using a metatranscriptomic approach

**DOI:** 10.1007/s10482-018-1040-x

**Published:** 2018-02-19

**Authors:** Loo Wee Chia, Bastian V. H. Hornung, Steven Aalvink, Peter J. Schaap, Willem M. de Vos, Jan Knol, Clara Belzer

**Affiliations:** 1 Laboratory of Microbiology, Wageningen University & Research, Stippeneng 4, 6708 WE Wageningen, The Netherlands; 2 Laboratory of Systems and Synthetic Biology, Wageningen University & Research, Stippeneng 4, 6708 WE Wageningen, The Netherlands; 3 RPU Immunobiology, Faculty of Medicine, University of Helsinki, Haartmaninkatu 3, 00290 Helsinki, Finland; 4 Nutricia Research, Uppsalalaan 12, 3584 CT Utrecht, The Netherlands

**Keywords:** Butyrate, Cross feeding, Keystone species, Microbiome, Mucin, Transcriptional regulation, *Verrucomicrobia*

## Abstract

Host glycans are paramount in regulating the symbiotic relationship between humans and their gut bacteria. The constant flux of host-secreted mucin at the mucosal layer creates a steady niche for bacterial colonization. Mucin degradation by keystone species subsequently shapes the microbial community. This study investigated the transcriptional response during mucin-driven trophic interaction between the specialised mucin-degrader *Akkermansia muciniphila* and a butyrogenic gut commensal *Anaerostipes caccae*. *A. muciniphila* monocultures and co-cultures with non-mucolytic *A. caccae* from the *Lachnospiraceae* family were grown anaerobically in minimal media supplemented with mucin. We analysed for growth, metabolites (HPLC analysis), microbial composition (quantitative reverse transcription PCR), and transcriptional response (RNA-seq). Mucin degradation by *A. muciniphila* supported the growth of *A. caccae* and concomitant butyrate production predominantly via the acetyl-CoA pathway. Differential expression analysis (DESeq 2) showed the presence of *A. caccae* induced changes in the *A. muciniphila* transcriptional response with increased expression of mucin degradation genes and reduced expression of ribosomal genes. Two putative operons that encode for uncharacterised proteins and an efflux system, and several two-component systems were also differentially regulated. This indicated *A. muciniphila* changed its transcriptional regulation in response to *A. caccae*. This study provides insight to understand the mucin-driven microbial ecology using metatranscriptomics. Our findings show that the expression of mucolytic enzymes by *A. muciniphila* increases upon the presence of a community member. This could indicate its role as a keystone species that supports the microbial community in the mucosal environment by increasing the availability of mucin sugars.

**Electronic supplementary material:**

The online version of this article (10.1007/s10482-018-1040-x) contains supplementary material, which is available to authorized users.

## Introduction

The bacterial assembly at the mucosal layer of the human gastrointestinal tract is associated with gut health and disease (Ouwerkerk et al. 2013; Tailford et al. 2015). Although the microbial composition of the healthy mucosa has not been properly defined, it has been observed that strong deviations in the mucosal microbiota are associated with inflammatory bowel disease (IBD) (Kostic et al. 2014) and irritable bowel syndrome (IBS) (Lopez-Siles et al. 2014). At this mucosal site, host-produced mucin glycans and bioactive compounds collectively exert a selective pressure that enriches for a sub-population of mucosa-associated bacteria (Koropatkin et al. 2012; Ouwerkerk et al. 2013; Schluter and Foster 2012). Mucins are large and complex glycoproteins consisting of a protein core that is rich in proline, threonine and serine moieties, to which oligosaccharides are attached (Tailford et al. 2015). Mucins can function as an indigenous prebiotic in which only specialised members of intestinal microbiota are able to utilise it as the substrate for growth (Marcobal et al. 2013; Ouwehand et al. 2005; Tailford et al. 2015).

The intestinal symbiont, *Akkermansia muciniphila* is the sole human intestinal representative of the phylum *Verrucomicrobia* (de Vos 2017). *A. muciniphila* has adapted to mucosal environment in the gut (Derrien et al. 2008). The genome of *A. muciniphila* is equipped with an arsenal of mucin-degrading enzymes including proteases, glycosyl hydrolases (GH), and sulfatases (Derrien et al. 2016; van Passel et al. 2011). The mucin-degrading capacity and oxygen tolerance of *A. muciniphila* render it a key species in the mucosal niche (Ouwerkerk et al. 2016). This specialised mucin-degrading bacterium is detected at high prevalence (over 96%) in healthy Western adults (Collado et al. 2007; Derrien et al. 2008; Shetty et al. 2016). The abundance of *A. muciniphila* in the gut microbiota is inversely correlated with syndromes such as IBDs (both Crohn’s disease and ulcerative colitis) (Png et al. 2010), appendicitis (Swidsinski et al. 2011) and obesity (Everard et al. 2013). Furthermore, the potential therapeutic role of *A. muciniphila* has been demonstrated in mice by remedying symptoms of obesity and diabetes (Plovier et al. 2017) as well as alcoholic liver disease (Grander et al. 2017).

In addition to the health-promoting role of *A. muciniphila* via immune modulation, the extracellular mucin degradation by this bacterium could provide growth benefits to community members via trophic interactions (Belzer et al. 2017; Belzer and de Vos 2012; Derrien et al. 2016). Several in vitro studies have demonstrated the butyrogenic effect of complex carbohydrates via cross-feeding between glycan-degrading bifidobacteria and butyrogenic bacteria (Belenguer et al. 2006; De Vuyst and Leroy 2011; Falony et al. 2006; Rios-Covian et al. 2015; Riviere et al. 2015; Schwab et al. 2017). In the mucosal environment, mucolytic bacteria such as *A. muciniphila*, *Bacteroides* spp. and *Ruminococcus* spp. as well as butyrogenic members of the family *Lachnospiraceae* (also known as *Clostridium* cluster *XIVa*) and *Ruminococcaceae* (also known as *Clostridium* cluster *IV*) are enriched (Nava et al. 2011; Van den Abbeele et al. 2013). However, no mucolytic capacities of these butyrogenic bacteria are known, which suggested potential metabolic cross-feeding between the microbial groups. Butyrate production in the vicinity of epithelial cells is suggested to be important in maintaining gut health (Koh et al. 2016; Louis and Flint 2017).

In a previous study (Belzer et al. 2017), we showed that mucin degradation by *A. muciniphila* yields short chain fatty acids (SCFAs) and mucin-derived monosaccharides that support the growth and concomitant butyrate production of non-mucolytic butyrogens. In this paper, we used metatranscriptomics (RNA-seq) to study the molecular response of mucin-directed trophic interaction between *A. muciniphila* and an abutyrogenic bacterium from the family *Lachnospiraceae* (*Anaerostipes caccae*) which possesses metabolic capacity to convert acetate and lactate into butyrate (Duncan et al. 2004) and shows frequent occurrence at the mucosal niche (Nava et al. 2011; Van den Abbeele et al. 2013). We demonstrated the use of metatranscriptomics as an explorative approach to study the expressional changes of *A. muciniphila* in response to a community member. Notably, we showed that *A. muciniphila* increased its mucolytic activity to sustain the community.

## Materials and methods

### Bacterial strains and growth conditions

All bacteria were grown in anaerobic serum bottles sealed with butyl-rubber stoppers at 37 °C with N_2_:CO_2_ (80:20 ratio) in the headspace at 1.5 atm. Bacterial pre-cultures were prepared by overnight growth in: minimal media supplemented with type III hog gastric mucin (Sigma-Aldrich, St. Louis, USA) for *A. muciniphila* Muc^T^ (ATCC BAA-835)(Derrien et al. 2004), and peptone yeast glucose (PYG) medium for *A. caccae* L1-92 (DSM 14662) (Schwiertz et al. 2002). Growth was measured by spectrophotometer as optical density at 600 nm (OD_600_) (OD600 DiluPhotometer™, IMPLEN, Germany).

### Co-culture experiment

**Fig. 1:**
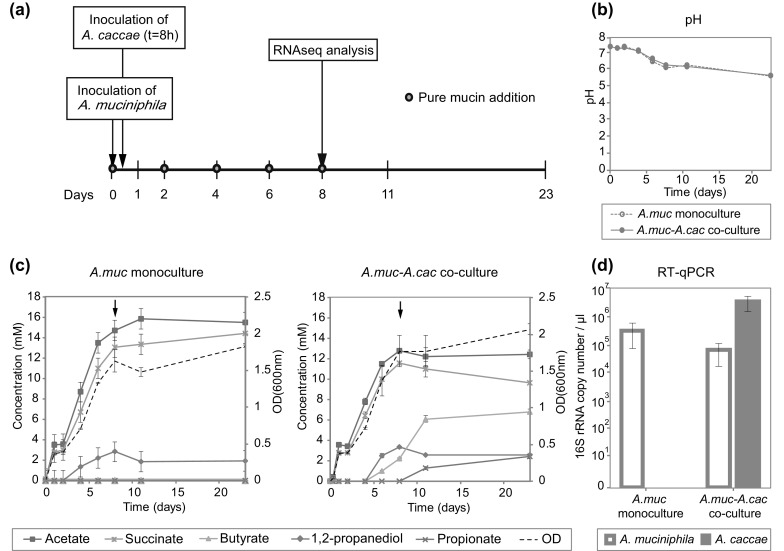
**a** Schematic overview of the interval-fed batch culture setup. *A. muciniphila* was inoculated at t = 0 h followed by *A. caccae* at t = 8 h to ensure substrate availability for butyrogen via extracellular mucin degradation by *A. muciniphila*. Limited amounts of pure mucin, 0.15% (v/v) were supplemented at 2 days intervals to maintain the abundance of *A. muciniphila* and to support the emergence of *A. caccae*. A sample for RNA-seq analysis was collected on day 8. **b** The pH and **c** metabolite profile of monocultures and co-cultures of the interval-fed batch culture, with arrow showing day 8. **d** Quantification of microbial composition on day 8 by RT-qPCR targeting 16S rRNA on total RNA. Error bars indicate the standard deviation of biological duplicates

Co-culture experiments were performed in minimal media (Plugge 2005) supplemented with purified hog gastric mucin (Miller and Hoskins 1981). Culture conditions were established as previously described (Belzer et al. 2017). *A. muciniphila* was inoculated at 1 × 10^6^ cells to mucin media followed by 8 h of incubation to allow accumulation of metabolites. Subsequently, 1 × 10^6^ cells of *A. caccae* (*A.muc*-*A.cac* co-cultures) were added to the *A. muciniphila* cultures. Cells were washed twice with phosphate-buffered saline (PBS) before addition to the co-cultures to prevent carryover of metabolites from the pre-cultures. Purified mucin (1.25 g l^−1^) was added to the media every 48 h. A schematic setup of the experiment is depicted in Fig. 1a. Cultures were sampled at 0, 1, 2, 4, 6, 8, 11, and 23 days for metabolites analysis. For transcriptomic analysis at day 8, bacteria pellets were preserved in Trizol^®^ reagent (Invitrogen, Carlsbad, CA, USA) at − 20 °C storage till further RNA purification.

### High-performance liquid chromatography (HPLC)

For metabolites analysis, 1 ml of bacterial culture was centrifuged and the supernatant was stored at − 20 °C until HPLC analysis. Crotonate was used as the internal standard, and the external standards were lactate, formate, acetate, propionate, isobutyrate, butyrate, citrate, malate, succinate, fumarate, 1,2-propanediol, methanol, ethanol, 2-propanol, lactose, *N*-acetylgalactosamine (GalNAc), *N*-acetylglucosamine (GlcNAc),glucose, and galactose. Substrates conversion and products formation were measured with a Spectrasystem HPLC (Thermo Scientific, Breda, the Netherlands) equipped with a Hi-Plex-H column (Agilent, Amstelveen, the Netherlands) for the separation of organic acids and carbohydrates. A Hi-Plex-H column performs separation with diluted sulphuric acid on the basis of ion-exchange ligand-exchange chromatography. Measurements were conducted at a column temperature of 45 °C with an eluent flow of 0.8 ml min^−1^ flow of 0.01 N sulphuric acid. Metabolites were detected by refractive index (Spectrasystem RI 150, Thermo, Breda, the Netherlands).

### RNA purification

Total RNA was isolated by a method combining the Trizol^®^ reagent and the RNeasy Mini kit (QIAGEN GmbH, Hilden, Germany) as described previously (Chomczynski 1993; Zoetendal et al. 2006). Four microliter of *p*-mercaptoethanol and 0.4 ml of buffer RLT were added to 1 ml of Trizol^®^ reagent containing the bacterial pellet. The mixture was transferred to a tube containing 0.8 g of glass beads (diameter 0.1 mm), followed by three times of bead beating for 1 min at 5.5 ms^−1^ with ice cooling steps in between. Subsequently, 0.2 ml of ice-cold chloroform was added. The solution was mixed gently followed by centrifugation at 12,000×*g* for 15 min at 4 °C. The RNA isolation was continued with the RNA clean-up according to the manufacturer’s instructions for the RNeasy Mini kit. Genomic DNA was removed by an on-column DNase digestion step during RNA purification (DNase I recombinant, RNase-free, Roche Diagnostics GmbH, Mannheim, Germany). Yield and RNA quality was assessed using the Experion™ RNA StdSens Analysis Kit in combination with the Experion™ System (Bio-Rad Laboratories Inc., Hercules, CA, USA).

### Quantitative reverse transcription PCR (RT-qPCR)

cDNA was synthesised using the ScriptSeq v2 RNA-Seq library preparation kit (Epicentre, Madison, WI, USA) according to the manufacturer’s instructions followed by purification using CleanPCR (CleanNA, the Netherlands). The cDNA was analysed by quantitative real-time PCR. Primers targeting 16S rRNA gene of *A. muciniphila* (AM1 5′-CAGCACGTGAAGGTGGGGAC-3′ and AM2 5′-CCTTGCGGTTGGCTTCAGAT-3′) (Collado et al. 2007), and *A. caccae* (OFF2555 5′-GCGTAGGTGGCATGGTAAGT-3′ and OFF2556 5′-CTGCACTCCAGCATGACAGT-3′) (Veiga et al. 2010) were used for quantification. Standard template DNA was prepared by 16S rRNA gene amplification of each bacterium with primers 27F (5′-AGAGTTTGATCCTGGCTCAG-3′) and 1492R (5′-GGTTACCTTGTTACGACTT-3′). Standard curves were prepared with nine standard concentrations from 10^0^ to 10^8^ gene copies μl^−1^. qPCR was performed in technical triplicate with iQ SYBR Green Supermix (Bio-Rad) in a total volume of 10 μl with primers at 500 nM in 384-well plates sealed with optical sealing tape. Amplification was performed with an iCycler (Bio-Rad) with the following protocol: one cycle of 95 °C for 10 min, 35 cycles of 95 °C for 15 s, 60 °C for 20 s, and 72 °C for 30 s each, one cycle of 95 °C for 1 min, one cycle of 60 °C for 1 min, and a stepwise increase of the temperature from 60 to 95 °C (at 0.5 °C per 5 s) to obtain melt curve data. Data were analysed using the Bio-Rad CFX Manager 3.0.

### Transcriptome sequencing (RNA-seq)

Total RNA samples were further processed by Baseclear for RNA-seq (Leiden, the Netherlands). Depletion of ribosomal RNA was performed using the Ribo-Zero™ Kit for bacteria (Epicentre, Madison, WI, USA) followed by quality monitoring using the Agilent 2100 BioAnalyzer system. Library construction for whole transcriptome sequencing was done using the TruSeq Stranded mRNA Library Prep Kit (Illumina, USA). The barcoded cDNA libraries were analysed using BioAnalyzer and were subsequently pooled and sequenced. Single read 50 bp sequencing was performed on two lanes using the Illumina HiSeq 2500 platform.

### Transcriptome analysis

The RNA-seq data was pre-processed for quality control. Ribosomal RNA was removed with SortMeRNA v2.0 (Kopylova et al. 2012) followed by all TruSeq adapters removal with Cutadapt v1.1.a (Martin 2011). Next, quality trimming was performed using Sickle v1.33 (Joshi and Fass 2011) with a score of 30 for threshold indicating a base calling confidence of 99.9%. Reads trimmed to a length < 50 bp were removed. Reads were subsequently mapped to the relevant bacterial genomes with Bowtie2 v0.6 (Langmead and Salzberg 2012) using default settings. HTSeq v0.6.1p1 was used to determine the read count for each protein coding region (Anders et al. 2015). All these steps were performed within a local Galaxy environment (Afgan et al. 2016). More detailed information about the data analysis can be found in Table S1. Non-mapping reads of the two samples with the lowest mapping rate (both of the *A. muciniphila* monocultures) were collapsed to unique reads with the fastx toolkit version 0.0.14 (http://hannonlab.cshl.edu/fastx_toolkit/). A blast search (with standard parameters, except for an e-value of 0.0001) of these unique reads was performed against the NCBI NT database (download 22.01.2014), against the human microbiome (download 08.05.2014), the NCBI bacterial draft genomes (download 23.01.2014), and the human genome (download 30.12.2013, release 08.08.2013, NCBI *Homo sapiens* annotation release 105). Taxonomy was estimated with a custom version of the LCA algorithm as implemented in MEGAN (Huson et al. 2011). Default parameters were used with the customization that only hits exceeding a bitscore of 50 and a length of more than 25 nucleotides were considered. 98% of the non-mapping reads were not classified, with *Akkermansia* accounting for 1.15% of the classified reads (Table S2). Differential gene expression was assessed using DESeq2 (Love et al. 2015). Raw RNA-seq sequence files can be accessed at the European Nucleotide Archive under accession numbers ERR1907419, ERR1907420, ERR1907423, and ERR1907424.

### Carbohydrate-active enzymes (CAZymes) prediction

CAZymes were predicted with dbCAN version 3.0 (Yin et al. 2012), transmembrane domains with TMHMM version 2.0c (Krogh et al. 2001) and signal peptides with signalP 4.1 (Petersen et al. 2011).

## Results

### Metabolite profile of *A. muciniphila* monocultures and co-cultures with *A. caccae*

Co-culturing of *A. muciniphila* and *A. caccae* was performed followed by RT-qPCR, HPLC and metatranscriptomic analysis. The metabolites detected in the cultures were comparable with previous findings (Belzer et al. 2017). *A. muciniphila* grown as monoculture produced acetate, succinate and 1,2-propanediol as the major metabolites from pure mucin degradation (Fig. 1c). On day 8 the *A.muc*-*A.cac* co-cultures yielded around 2 mM butyrate and a low amount of propionate was detected (Fig. 1c). The mucin sugars (galactose, GalNAc, and GlcNAc) were below the detection limit of 0.5 mM.

### The transcriptomes of *A. muciniphila* monocultures and co-cultures with *A. caccae*

Transcriptomic samples were analysed on day 8 of the interval-fed batch cultures, when the major metabolites were accumulated (Fig. 1c) and a stable bacterial composition was established (Belzer et al. 2017). On average 27 million reads were generated per sample, which is above the recommended sequence depth of 5–10 million reads for a single bacterial transcriptome (Haas et al. 2012). The detailed information about the data analysis can be found in Table S1. The RT-qPCR targeting 16S rRNA on total RNA showed an *A. muciniphila* to *A. caccae* ratio of 1:50 (Fig. 1d). On the other hand, the ratio of sequenced transcripts mapped to the genome of *A. muciniphila* versus *A. caccae* was 1:1 (Table S1).

### Differential expression between *A. muciniphila in* monocultures and co-cultures with *A. caccae*

**Fig. 2:**
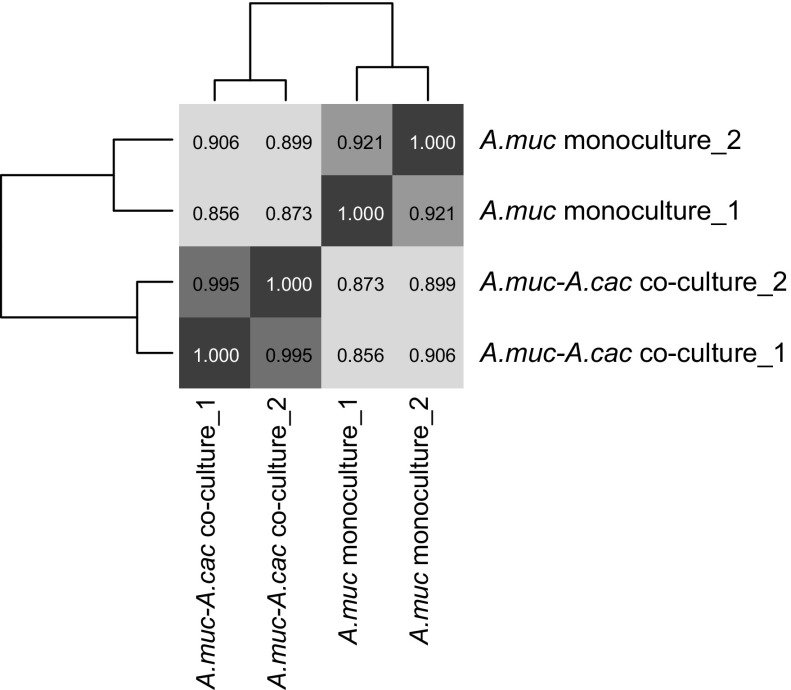
Hierarchical clustering showing the Pearson’s correlation of the transcriptome samples as calculated from *A. muciniphila* CDS count performed with Python 2.7.12 and SciPy version 0.17.1 (van der Walt et al. 2011)

The genome of *A. muciniphila* possesses a total of 2176 predicted protein-coding sequences (CDSs) (van Passel et al. 2011) of which 2137 (98%) were found to be expressed in this study (Table S3). Differential expression analysis (DESeq2) was performed to compare the gene expression of *A. muciniphila* in mono- and co-culture conditions. The overall transcriptional response differentiated between the mono- and co-cultures (Pearson’s correlation = 0.88 ± 0.02) (Fig. 2).

**Fig. 3:**
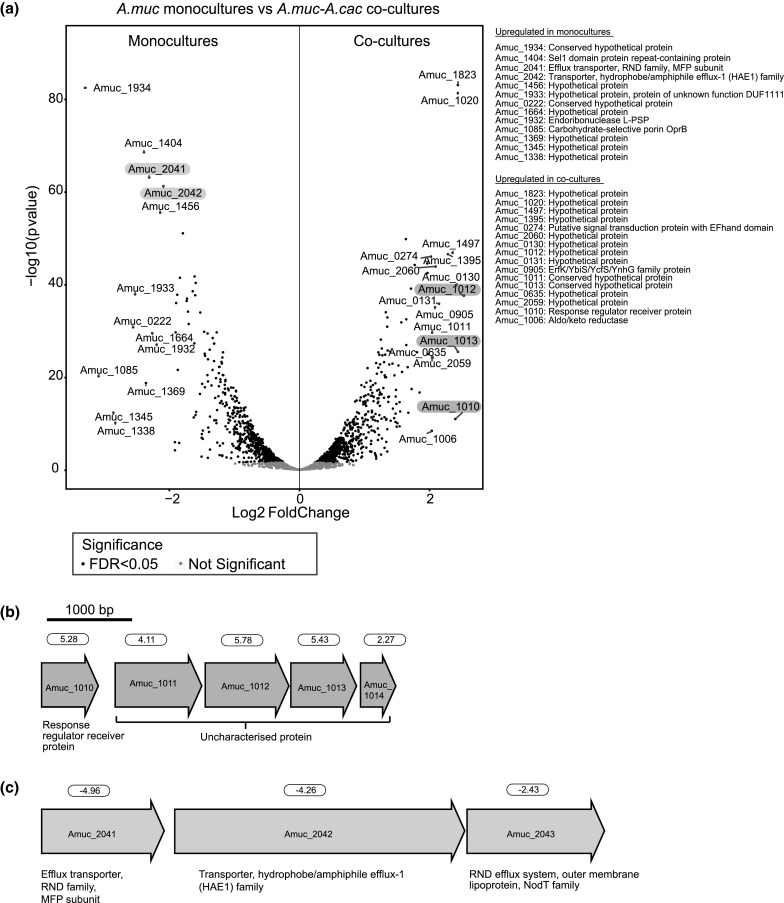
**a** Volcano plots showing p-values correlated to fold changes in gene expression of *A. muciniphila* observed in monocultures versus co-cultures with *A. caccae*. Positive fold changes indicate upregulation in co-cultures, and negative fold changes indicate upregulation in monocultures. Locus tags for genes with Log2 fold change > 2 (or fold change > 4) are labelled. **b** Response regulator and putative operon upregulated in the co-cultures. **c** Putative operon upregulated in the monocultures. Fold changes are listed above the respective genes

We used cut-offs of q < 0.05 and fold change > 2 for significantly regulated genes (Schurch et al. 2016). A total of 12% *A. muciniphila* genes were differentially regulated between mono- and co-cultures, with 148 upregulated genes and 132 downregulated genes (Table S3). Interestingly, two groups of contiguous genes were differentially regulated at high fold change (Fig. 3a). In the co-cultures, the upregulation of the annotated response regulator Amuc_1010 was coupled with the upregulation of a putative operon containing the genes Amuc_1011, Amuc_1012, Amuc_1013, and Amuc_1014 (Fig. 3b). Whereas, the putative operon consisting of Amuc_2041, Amuc_2042 and Amuc_2043 was downregulated in the co-cultures (Fig. 3c). Furthermore, several putative two-component systems were differentially expressed (Table 1).

**Table 1:** The differential expression of putative two-component systems in *A. muciniphila*

Locus tag	*A.muc*-*A.cac* co-culture		Function
	q value	Fold change	
Amuc_0311	< 0.05	1.96	Signal transduction histidine kinase, nitrogenspecific, NtrB
Amuc_0312	< 0.05	2.19	Two-component, sigma54 specific, transcriptional regulator, Fis family
Amuc_0827	< 0.05	1.44	Osmo-sensitive K^+^ channel signal transduction histidine kinase
Amuc_0828	< 0.05	1.74	Two-component transcriptional regulator, winged helix family
Amuc_1109	< 0.05	− 1.89	Histidine kinase
Amuc_1110	0.53	− 1.07	Two-component transcriptional regulator, winged helix family
Amuc_1727	0.63	1.06	Integral membrane sensor signal transduction histidine kinase
Amuc_1728	0.25	1.13	Two-component transcriptional regulator, winged helix family
Amuc_1010	< 0.05	5.28	Response regulator receiver protein

Negative values indicate upregulation in monocultures and positive values indicate upregulation in co-cultures

Gene ontology analysis (Table 2) showed overall increase expression of hydrolase activity, DNA recombination enzymes, and sulphuric ester hydrolase activity in the co-cultures whereas ribosome, structural constituent of ribosome and translation were downregulated. The list of *A. muciniphila* CAZymes is summarised in Table S4. The overall expression of glycosyl hydrolases was upregulated in the co-cultures. Signal peptides and transmembrane domains prediction showed putative extracellular activity for glycosyl hydrolases required for the degradation of mucin *O*-glycan chains including GH2, GH20, GH29, GH33, GH84, GH89, and GH98.

**Table 2:** Gene ontology (GO) analysis of the differentially regulated *A. muciniphila* genes (q < 0.05) in co-cultures

GO term	Total count in *A.muc* genome	Percentage upregulated	Percentage downregulated
*A.muc*-*A.cac* co-culture			
** GO:hydrolase activity, hydrolyzing O-glycosyl compounds**	**30**	**0.60**	**0.03**
**GO:DNA recombination**	**17**	**0.53**	**0.06**
**GO:sulphuric ester hydrolase activity**	**12**	**0.50**	**0.17**
*GO:transporter activity*	*27*	*0.22*	*0.52*
*GO:magnesium ion binding*	*16*	*0.19*	*0.44*
*GO:tRNA processing*	*11*	*0.18*	*0.55*
*GO:cytoplasm*	*66*	*0.17*	*0.48*
*GO:pyridoxal phosphate binding*	*20*	*0.15*	*0.45*
*GO:RNA binding*	*37*	*0.14*	*0.46*
*GO:GTP binding*	*20*	*0.10*	*0.55*
*GO:transferase activity*	*21*	*0.10*	*0.43*
*GO:tRNA aminoacylation for protein translation*	*24*	*0.08*	*0.71*
*GO:cellular amino acid metabolic process*	*12*	*0.08*	*0.50*
*GO:aminoacyl-tRNA ligase activity*	*25*	*0.08*	*0.72*
* GO:nucleotide binding*	*40*	*0.08*	*0.58*
* GO:intracellular*	*42*	*0.07*	*0.79*
*GO:NAD binding*	*15*	*0.07*	*0.33*
* GO:ribosome*	*50*	*0.02*	*0.88*
*GO:structural constituent of ribosome*	*55*	*0.02*	*0.89*
*GO:translation*	*57*	*0.02*	*0.88*

The list contains GO with total count in genome higher than 10 and absolute percentage difference higher than average value. GO with overall expression upregulated or downregulated in co-cultures are marked in bold and italic respectively

### Genes expression in relation to the metabolites production

We examined the transcripts of the co-cultures to reconcile the metabolite findings. The transcripts for *A. caccae* showed median of relative abundance around 0.005% and maximum value of 2.07%. The list of *A. caccae* genes is displayed in Table S5. It is reported that *A. caccae* metabolises acetate to butyrate by employing the most prevalent butyrate production pathway via acetyl-coenzyme A (CoA) (Vital et al. 2014). The relative abundances of all transcripts involved in the metabolism pathways are summarised in Table 3. Our data indicated that the majority of enzymes involved in the acetyl-CoA pathway were expressed at a relative abundance higher than 0.1%, with over 2% of total transcripts accounted for butyrate production. In addition, *A. caccae* possesses genomic capacity to synthesis butyrate by using 4-aminobutyrate or succinate as the precursor. However, the expression of this pathway was low, with the relative abundance of transcripts lower than 0.01%, indicating that acetyl-CoA was the dominant pathway.

**Table 3:** The relative abundance (%) of *A. caccae* transcripts for genes involved in butyrate synthesis pathway

Enzyme	Locus tag	Dup1	Dup2
**Interconversion of pyruvate to acetyl-CoA**			
Pyruvate dehyrogenase complex	ANACAC_01488	< 0.00	< 0.00
	ANACAC_01489	< 0.00	< 0.00
	ANACAC_01490	< 0.00	< 0.00
	ANACAC_01491	< 0.00	< 0.00
	ANACAC_01492	< 0.00	< 0.00
Formate C-acetyltransferase	ANACAC_01621	< 0.00	< 0.00
	ANACAC_00664	< 0.00	< 0.00
Pyruvate synthase	ANACAC_00834	1.83	1.85
**Interconversion of pyruvate to lactate**			
l-Lactate dehydrogenase	ANACAC_01148	0.01	0.01
	ANACAC_03769	0.02	0.02
**Acetyl-CoA pathway**			
Acetyl-CoA C-acetyltransferase	ANACAC_00256	0.34	0.37
Acetoacetyl-CoA reductase	ANACAC_00254	0.35	0.39
3-Hydroxybutyryl-CoA dehydratase	ANACAC_03496	0.01	0.02
	ANACAC_00255	0.21	0.23
Butyryl-CoA dehydrogenase	ANACAC_00252	0.50	0.50
	ANACAC_00253	0.54	0.56
	ANACAC_03492	0.00	0.00
Phosphate acetyltransferase	ANACAC_00344	0.13	0.15
Acetate kinase	ANACAC_00343	0.17	0.18
Butyryl-CoA:acetate CoA-transferase	ANACAC_01149	0.16	0.17
**4-Aminobutyrate/succinate pathway**			
Hydroxybutyrate dehydrogenase	ANACAC_00166	< 0.00	< 0.00
4-Hydroxybutyrate coenzyme A transferase	ANACAC_00165	< 0.00	< 0.00
4-Hydroxybutanoyl-CoA dehydratase	ANACAC_00167	< 0.00	< 0.00
	ANACAC_02698	< 0.00	< 0.00

### Nutrients interdependency between *A. muciniphila* and *A. caccae*

The genomes of *A. muciniphila* and *A. caccae* were inspected for B vitamins and amino acids auxotrophy to investigate potential nutrient interdependency. *A. muciniphila* lacked the upstream genes required for vitamin B12 biosynthesis including CbiL, CobG, CbiGF, CobF, CbiECA and CobAT. Complementarily, *A. caccae* was predicted to possess a complete vitamin B12 biosynthesis pathway (Table 4). However, no vitamin B12 transporter was found in the *A. caccae* genome. We found indications for aspartate auxotrophy of *A. caccae* (Table S6) however the bacterium was reported to grow in minimal defined media supplemented with glucose without additional nitrogen source (Belzer et al. 2017). Furthermore, *A. caccae* lacks the genes to synthesise the cofactor lipoate required for dehydrolipoate dehydrogenase, EC 1.8.1.4. The different enzyme complexes containing this enzyme are involved in citrate cycle, glycine, serine, and threonine metabolism, and valine, leucine, and isoleucine degradation. Nevertheless, *A. caccae* could acquire lipoate via salvage pathway and we observed the upregulation of lipoate biosynthesis by *A. muciniphila* in co-cultures.

**Table 4:** Genomic prediction of B vitamins biosynthesis (presence = 1 and absence = 0) based on the combination of essential functional roles by Magnusdottir et al. (2015)

	B7	B12	B9	B3	B5	B6	B2	B1
	Biotin	Cobalamin	Folate	Niacin	Pantothenate	Pyridoxin	Riboflavin	Thiamin
*Akkermansia muciniphila* Muc^T^	1	0	1	1	1	1	1	1
*Anaerostipes caccae* L1-92	0	1	1	1	1	1	1	1

## Discussion

In this study, we demonstrated the use of metatranscriptomics as an explorative approach to decipher bacterial interaction in the mucosal environment. Two representative mucosa-associated species, namely *A. muciniphila* and *A. caccae*, were used to show the ecological dependency between a mucin degrader and a butyrate producer. Importantly, this study revealed changes in the expression of genes involved in host-glycan catabolism and trophic interactions between the gut commensals. This interplay leads to the formation of butyrate in the mucosal layer that is proposed to be beneficial to the host (Koh et al. 2016; Louis and Flint 2017).

In the presence of *A. caccae*, *A. muciniphila* upregulated mucin-degrading genes involved in hydrolase and sulphuric ester hydrolase activity. The majority of these mucin-degrading enzymes were predicted to function in the extracellular compartment (Ottman et al. 2016), which could lead to the degradation of oligosaccharide chains consisting of GalNAc, GlcNAc, mannose, galactose, fucose and sialic acid (Moran et al. 2011). Previous work demonstrated that *A. caccae* as well as *Eubacterium hallii* and *Faecalibacterium prausnitzii* could utilise the mucin-derived sugars including galactose, mannose and GlcNAc for growth (Belzer et al. 2017; Lopez-Siles et al. 2012). The fermentation of these monosaccharides results in butyrate production. Since both *A. muciniphila* and the butyrate-producer rely on the uptake of mucin-derived sugars for growth in our model, a higher extracellular concentration of *A. muciniphila*-derived mucolytic enzymes could contribute to substrate availability in the community. Concurrently, *A. muciniphila* showed downregulation of ribosomal genes in the co-cultures, which implied a lower growth rate of *A. muciniphila*. The qPCR results of genomic 16S rRNA gene ratio from a previous publication on extracted DNA showed a *A.muciniphila* to *A. caccae* ratio of 100:1 (Belzer et al. 2017). In this study, the ratio of 16SrRNA in total RNA samples quantified by RT-qPCR showed a *A. muciniphila* to *A. caccae* ratio of 1:50, whereas, the sequenced transcripts ratio was 1:1. The discrepancy could be the result of differential expression between ribosomal and messenger RNA. Note that total RNA could contain 95–99% of ribosomal RNA (Zoetendal et al. 2006) and that the number of ribosomes per cell correlates with the growth rate (Fegatella et al. 1998). In addition, *A. muciniphila* and *A. caccae* contain 3 and 12 copies of the rRNA operon, respectively. Taken together, these results indicate that *A. muciniphila* dominated in terms of cells number but *A. caccae* showed proportionally higher growth rate and transcriptional activity.

**Fig. 4:**
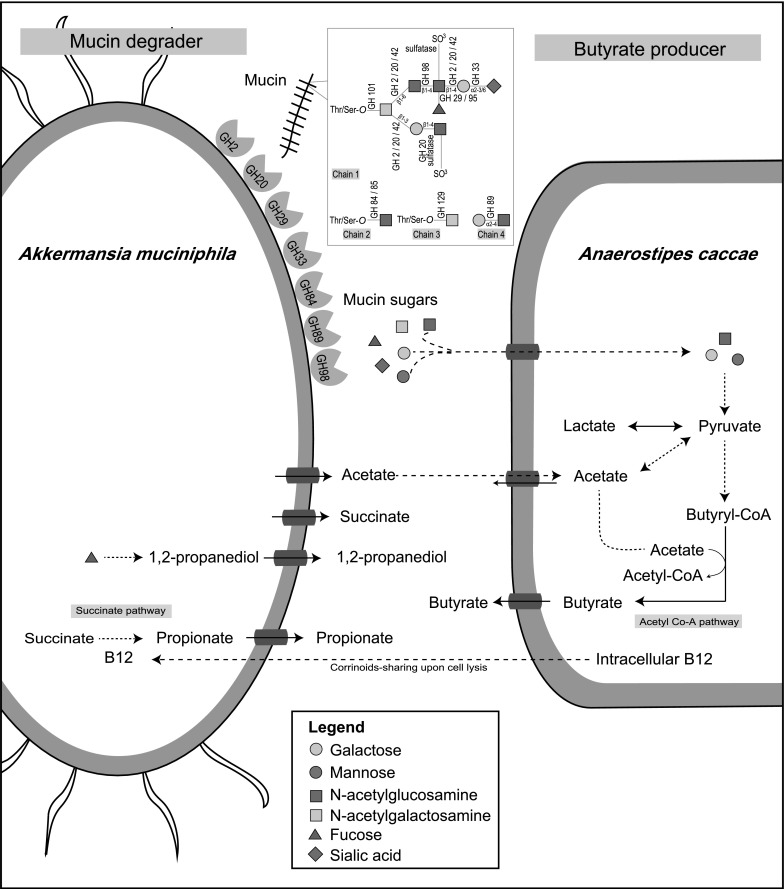
Schematic representation of mucin-driven trophic interaction between *A. muciniphila* and *A. caccae*. *A. muciniphila* degrades oligosaccharides chain of mucin by extracellular glycosyl hydrolases. The structure for *O*-linked glycan chains and CAZymes action sites are adapted from Tailford et al. (2015). Chain 1 is a hypothetical mucin glycan chain, chain 2 is *O*-GlcNAc often found on other glycoproteins, chain 3 (Tn antigen) and chain 4 are found in gastro-duodenal mucin. In addition, mannose could be released from degradation of *N*-linked glycan chains. *A. caccae* utilises some of the mucin-derived sugars (galactose, mannose and GlcNAc) and acetate released by *A. muciniphila* for growth and concomitant butyrate production

The co-culturing of two representative mucosa-associated bacteria has demonstrated the major pathways for intestinal SCFAs biosynthesis. The overview of this mucin-directed trophic interaction is shown in Fig. 4. *A. caccae* cross-fed on a part of the mucin sugars liberated by *A. muciniphila* for central metabolism. In addition, *A. caccae* can incorporate *A. muciniphila*-derived acetate for butyrate production via butyryl-CoA:acetate CoA-transferase enzyme(Duncan et al. 2004; Louis and Flint 2009; Louis and Flint 2017). Moreover, *A. muciniphila* could benefit from the corrinoids released by *A. caccae* (Degnan et al. 2014). Pseudo-vitamin B12 from *E. hallii* could activate the propionate production by *A. muciniphila* via the succinate pathway (Belzer et al. 2017). A low level of propionate was detected after day 8 in *A.muc*-*A.cac* co-cultures (Belzer et al. 2017). Propionate is likely produced by *A. muciniphila* because *A. caccae* is not known to produce propionate and it does not possess the genes involved in the known propionate biosynthesis pathways i.e. the succinate, acrylate, and propanediol pathways (Louis and Flint 2017). Nevertheless, *A. caccae* is predicted to synthesise vitamin B12 but lacked a vitamin B12 transporter. Upon cell lysis, the release of cellular vitamin B12 by *A. caccae* could facilitate methylmalonyl-CoA mutase enzymes (Amuc_1983 and Amuc_1984) of *A. muciniphila* to produce propionate (Degnan et al. 2014). The upregulation of cobalamin-dependent methylmalonyl-CoA mutase genes in monocultures indicated an attempt by the organism to activate the propionate production pathway in the absence of the essential cofactor (Fig. S1), as the conversion of methylmalonyl-CoA to propionyl-CoA is thermodynamically favourable (Dimroth and Schink 1998). The exergonic decarboxylation of methylmalonyl-CoA could be coupled to sodium ion export to extracellular space for the establishment of a proton gradient via a sodium-proton antiporter to generate ATP (Ottman et al. 2017a).

Interestingly, two putative operons and several two-component systems were differentially regulated, indicating the mode of transcriptional regulation by *A. muciniphila* in response to *A. caccae*. A previous study has demonstrated that the presence of one organism is often associated with transcriptional changes in the other (Plichta et al. 2016). In the co-culture with *A. caccae*, *A. muciniphila* downregulated a putative operon consisting of Amuc_2041 (efflux transporter, RND family, MFP subunit), Amuc_2042 (transporter, hydrophobe/amphiphile efflux-1 (HAE1) family) and Amuc_2043 (RND efflux system, outer membrane lipoprotein, NodT family). The membrane fusion protein (MFP) is described as a component of drug resistance, nodulation, and the cell division (RND) family involved in the transportation of drug molecules (Anes et al. 2015). HAE1 is involved in toxin production and resistance processes (Anes et al. 2015). The outer membrane lipoproteins from the NodT family are predicted to primarily export small molecules rather than proteins. This efflux system was reported to play a role in multidrug resistance of Gram-negative bacteria such as *Escherichia coli* and *Pseudomonas aeruginosa* (Nikaido and Takatsuka 2009). A similar resistance mechanism could be employed by the Gram-negative *A. muciniphila*, and this study suggested the down-tuning of the efflux pump expression in the presence of a community member.

The annotated response regulator Amuc_1010 and the adjacent predicted operon consisting of Amuc_1011, Amuc_1012, Amuc_1013, and Amuc_1014, were upregulated in the co-cultures. Amuc_1010 is likely not a two-component system as it encoded only for the LytTR DNA-binding domain without the CheY-like receiver domain. Amuc_1010 could be autoregulatory as *cis*-acting regulatory elements were predicted at its upstream region using MEME (Bailey et al. 2009) (data not shown). Amuc_1011, Amuc_1012, Amuc_1013, and Amuc_1014 were annotated as uncharacterised proteins, and Amuc_1011 was predicted as an outer membrane protein (Ottman et al. 2016). Further research is needed to investigate this interesting gene cluster with unidirectional arrangement and a short intercistronic region that could likely be co-transcribed. The upregulation of the outer membrane protein could be associated with host colonization, persistence and immunomodulation (Galdiero et al. 2012). A recent study showed that an immune-stimulatory outer membrane protein of *A. muciniphila* (Amuc_1100) (Ottman et al. 2017b) is able to ameliorate the metabolic symptoms of obese and diabetic mice (Plovier et al. 2017). However, Amuc_1100 was not found to be differentially regulated in this study.

In addition, *A. muciniphila* upregulated several two-component systems in the co-cultures. Two-component systems consist of a membrane bound sensor histidine kinase and a cytoplasmic response regulator, which are often encoded by adjacent genes, enable bacteria to response to changing environment by altering gene expression (Monedero et al. 2017). However, the roles of two-component systems in *A. muciniphila* grown in the co-cultures were not yet identified. Studies showed that they could be involved in the regulation of physiological processes in commensal bacteria, such as stress responses, regulation of metabolism, and resistance to antimicrobial peptides (Monedero et al. 2017). The gastrointestinal pathogen, enterohemorrhagic *E. coli* (EHEC), was reported to encode the two-component system FusKR. This system provides a growth advantage and modulates the expression of virulence genes upon sensing of fucose liberated by *Bacteroides thetaiotaomicron* during growth in media containing mucin (Pacheco et al. 2012). The metabolism of mucin-derived fucose by *A. muciniphila* yielded 1,2-propanediol (Ottman et al. 2017a). As such, fucose metabolism by *A. muciniphila* could confer colonization resistance against the fucose-dependent enteric pathogens (Pickard and Chervonsky 2015).

In conclusion, we demonstrated the use of metatranscriptomics to provide in-depth mechanistic understanding of bacterial interaction. The trophic interaction between mucosal keystone species *A. muciniphila* and *A. caccae* could result in beneficial butyrate production at close proximity to the host epithelium. We revealed the expressional changes of *A. muciniphila* in response to *A. caccae* and demonstrated the provider role of *A. muciniphila* by upregulating the mucolytic activity to sustain the community at the mucosa niche.

## Electronic supplementary material

Below is the link to the electronic supplementary material.

Supplementary material 1 (PDF 422 kb)

Supplementary material 2 (XLSX 314 kb)

Supplementary material 3 (DOCX 20 kb)
