# Supplementary figures and images for: Deciphering the trophic interaction between Akkermansia muciniphila and the butyrogenic gut commensal Anaerostipes caccae using a metatranscriptomic approach
Source: Antonie Van Leeuwenhoek. 2018 Feb 19;111(6):859–73. doi: 10.1007/s10482-018-1040-x (PMC5945754; doi:10.1007/s10482-018-1040-x)

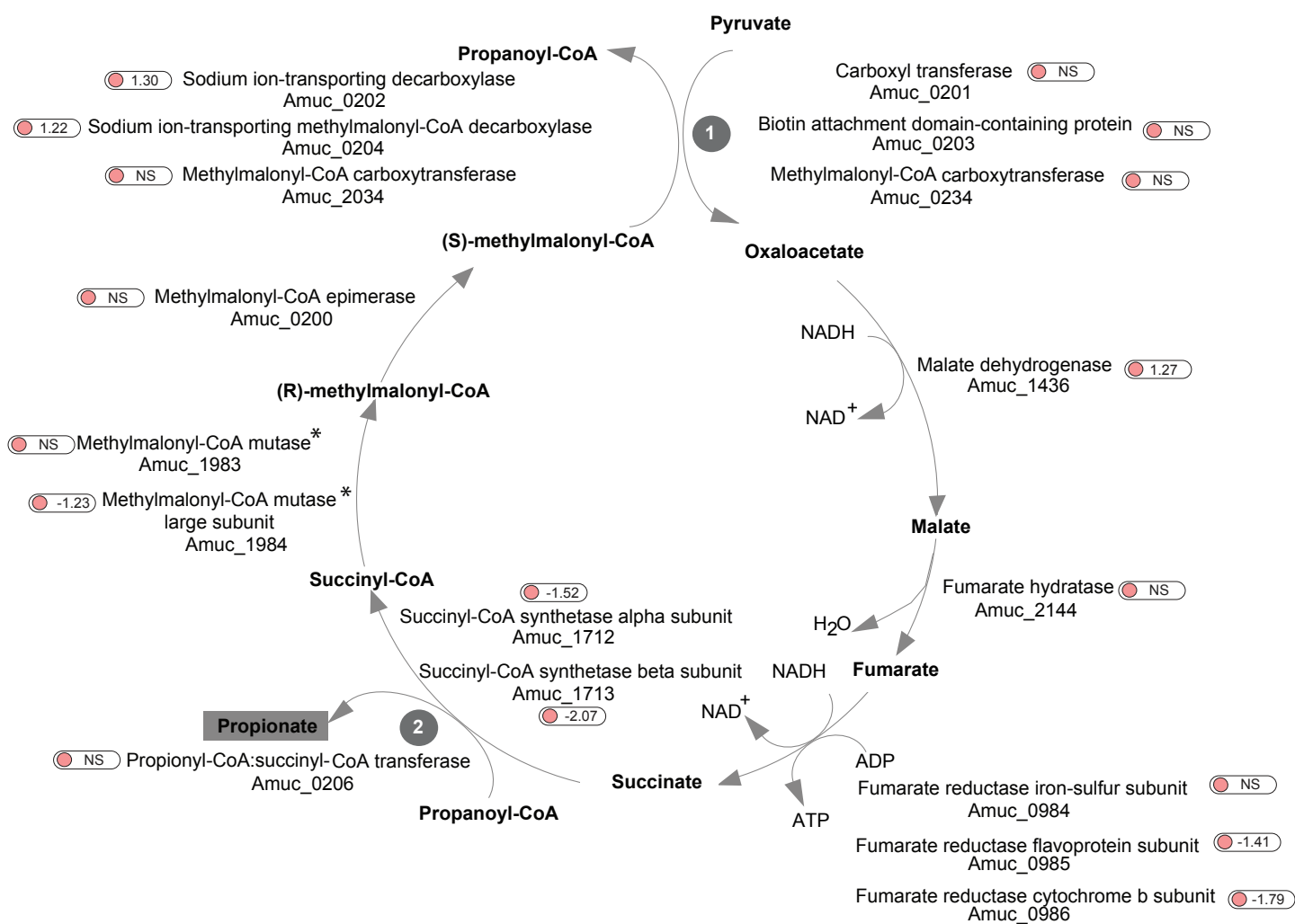

Supplement: Supplementary file 1 — Supplementary material 1 (PDF 422 kb) [file 10482_2018_1040_MOESM1_ESM.pdf]
