# Supplementary material for: Deciphering the trophic interaction between Akkermansia muciniphila and the butyrogenic gut commensal Anaerostipes caccae using a metatranscriptomic approach
Source: Antonie Van Leeuwenhoek. 2018 Feb 19;111(6):859–73. doi: 10.1007/s10482-018-1040-x (PMC5945754; doi:10.1007/s10482-018-1040-x)
Supplement: Supplementary file 3 — Supplementary material 3 (DOCX 20 kb) [file 10482_2018_1040_MOESM3_ESM.docx]

Table S1. The general features of RNA-seq data analysis.

|  | *A.muc*  monoculture | | *A.muc-A.cac*  co-culture | |
| --- | --- | --- | --- | --- |
|  | Duplicate 1 | Duplicate 2 | Duplicate 1 | Duplicate 2 |
| Total no. of reads | 30812972 | 27356734 | 26799549 | 22755873 |
| Average quality scores (Phred) | 37.72 | 37.72 | 38.73 | 38.72 |
| rRNA removed (%) | 6.4 | 4.1 | 3.9 | 4.4 |
| Adapters removed (%) | 4.3 | 4.7 | 5.4 | 5.4 |
| No. of reads after Cutadapt | 28826013 | 26236614 | 25748139 | 21756579 |
| Trimmed reads by Sickle (%) | 24.90 | 25.24 | 13.23 | 13.63 |
| No. of reads after Sickle (quality threshold=30; length threshold=50) | 21647836 | 19613896 | 22342387 | 18790981 |
| Total no. of reads mapped to the concatenated genome of *A.muciniphila &* butyrogens | NA | NA | 21433553 | 17850364 |
| Total no. of reads mapped to the genome of *A.muciniphila* | 17531380 | 15516414 | 10135288 | 8630425 |
| Total no. of reads mapped to the genome of butyrogens | NA | NA | 11298279 | 9219949 |
| Total no. of reads mapped to the concatenated genome of *A.muciniphila* & butyrogens (%) | NA | NA | 95.93 | 94.99 |
| Total no. of reads mapped to the genome of *A.muciniphila* (%) | 80.98 | 79.11 | 45.36 | 45.93 |
| Total no. of reads mapped to the genome of butyrogens (%) | NA | NA | 50.57 | 49.07 |
| Sum of % of reads mapped to the genomes | 80.98 | 79.11 | 95.93 | 95.00 |
| Total no. of reads mapped to the concatenated protein coding regions of *A.muciniphila &* butyrogens | NA | NA | 10286240 | 8436520 |
| Total no. of reads mapped to the protein coding regions of *A.muciniphila* | 8368120 | 8505786 | 3991352 | 3189066 |
| Total no. of reads mapped to the protein coding regions of butyrogens | NA | NA | 6294888 | 5247454 |
| Total no. of reads mapped to the protein coding regions | 8368120 | 8505786 | 10286240 | 8436520 |

NA denotes not available

Table S2. The blast result for the unique non-mapping reads from both of the *A. muciniphila* monocultures. (Available in excel file for supplementary table)

Table S3. Summary of the differentially expressed *A. muciniphila* CDS in the co-cultures with *A. caccae*.

|  | *A.muc-A.cac* co-culture |
| --- | --- |
| Total number of CDS | 2137 |
| Number of CDS with q>0.05 | 1003 |
| Number of CDS with q<0.05 |  |
| Upregulated |  |
| Fold change < 2 | 416 |
| 2 < Fold change < 4 | 132 |
| Fold change > 4 | 16 |
| Downregulated |  |
| Fold change < 2 | 438 |
| 2 < Fold change < 4 | 119 |
| Fold change > 4 | 13 |

Table S4. Differentially regulated CAZymes of *A. muciniphila* in *A.muc-A.cac* co-cultures (q<0.05). Signal peptide and transmembrane protein were predicted using SignalP and TMHMM respectively. (Available in excel file for supplementary table)

Table S5. The relative abundance and putative function of transcripts for *A. caccae*. (Available in excel file for supplementary table)

Table S6. The genomic prediction of amino acids biosynthesis.

| Amino acids | *Akkermansia muciniphila* Muc^T^ | *Anaerostipes caccae* L1-92 |
| --- | --- | --- |
| Alanine (Ala) | Complete | Complete |
| Arginine (Arg) | Complete | Complete |
| Asparagine (Asn) | Complete | Complete |
| Aspartic acid (Asp) | Complete | NA |
| Cysteine (Cys) | Complete | Complete |
| Glutamic acid (Glu) | Complete | Complete |
| Glutamine (Gln) | Complete | Complete |
| Glycine (Gly) | Complete | Complete |
| Histidine (His) | Complete | Complete |
| Isoleucine (Ile) | Complete | Complete |
| Leucine (Leu) | Complete | Complete |
| Lysine (Lys) | Complete | Complete |
| Methionine (Met) | Complete | Complete |
| Phenylalanine (Phe) | NA | NA |
| Proline (Pro) | Complete | Complete |
| Serine (Ser) | Complete | Incomplete |
| Threonine (Thr) | NA | Complete |
| Tryptophan (Trp) | Complete | Complete |
| Tyrosine (Tyr) | Incomplete | Incomplete |
| Valine (Val) | Complete | Complete |
| Selenocysteine (Sec) | Incomplete | Complete |
| Pyrrolysine (Pyl) | Incomplete | Incomplete |
| Homocysteine | Complete | Incomplete |
| Homoserine | Complete | Complete |
| Ornithine | Complete | Complete |

NA denotes not available

FigS1. *A. muciniphila* pathway for pyruvate fermentation to propionate (succinate pathway). Fold changes for differentially expressed genes (q <0.05) comparing *A. muciniphila* in monoculture and co-cultures with *A. caccae* are listed next to the respective genes. Negative values indicate upregulation in monocultures, positive values indicate upregulation in co-cultures and NS indicates non-significant regulation with q >0.05. Figure adapted from MetaCyc ([Caspi et al. 2014](#_ENREF_8)) with curation.
